# Unraveling the Chicken Meat Volatilome with Nanostructured Sensors: Impact of Live and Dehydrated Insect Larvae Feeding

**DOI:** 10.3390/s24154921

**Published:** 2024-07-29

**Authors:** Dario Genzardi, Estefanía Núñez Carmona, Elisabetta Poeta, Francesco Gai, Immacolata Caruso, Edoardo Fiorilla, Achille Schiavone, Veronica Sberveglieri

**Affiliations:** 1 Institute of Bioscience and Bioresources (CNR-IBBR), National Research Council, Via J.F. Kennedy, 17/i, 42124 Reggio Emilia, Italy; dario.genzardi@ibbr.cnr.it (D.G.); immacolata-caruso@cnr.it (I.C.); veronica.sberveglieri@ibbr.cnr.it (V.S.); 2 Department of Engineering “Enzo Ferrari”, University of Modena and Reggio Emilia, Via Pietro Vivarelli 10, 41125 Modena, Italy; 3 Department of Life Sciences, University of Modena and Reggio Emilia, Via J.F. Kennedy, 17/i, 42124 Reggio Emilia, Italy; 4 Institute of Sciences of Food Productions (CNR-ISPA), National Research Council Largo Paolo Braccini, 2, 10095 Grugliasco, Italy; francesco.gai@ispa.cnr.it (F.G.); achille.schiavone@unito.it (A.S.); 5 Department of Veterinary Sciences, University of Turin, Largo Paolo Braccini, 2, 10095 Grugliasco, Italy; edoardo.fiorilla@unito.it; 6 Nano Sensor System srl (NASYS), Via Alfonso Catalani 9, 42124 Reggio Emilia, Italy

**Keywords:** nanomaterials, MOX sensors, nanotechnologies, IoT applications, insect feeding, food quality

## Abstract

Incorporating insect meals into poultry diets has emerged as a sustainable alternative to conventional feed sources, offering nutritional, welfare benefits, and environmental advantages. This study aims to monitor and compare volatile compounds emitted from raw poultry carcasses and subsequently from cooked chicken pieces from animals fed with different diets, including the utilization of insect-based feed ingredients. Alongside the use of traditional analytical techniques, like solid-phase microextraction combined with gas chromatography-mass spectrometry (SPME-GC-MS), to explore the changes in VOC emissions, we investigate the potential of S3+ technology. This small device, which uses an array of six metal oxide semiconductor gas sensors (MOXs), can differentiate poultry products based on their volatile profiles. By testing MOX sensors in this context, we can develop a portable, cheap, rapid, non-invasive, and non-destructive method for assessing food quality and safety. Indeed, understanding changes in volatile compounds is crucial to assessing control measures in poultry production along the entire supply chain, from the field to the fork. Linear discriminant analysis (LDA) was applied using MOX sensor readings as predictor variables and different gas classes as target variables, successfully discriminating the various samples based on their total volatile profiles. By optimizing feed composition and monitoring volatile compounds, poultry producers can enhance both the sustainability and safety of poultry production systems, contributing to a more efficient and environmentally friendly poultry industry.

## 1. Introduction

Population growth, urbanization, and the growth of the middle class have increased the global demand for food, and in particular for sources of animal protein. In Europe, as in many parts of the world, poultry consumption constitutes a substantial portion of dietary protein intake and serves as a cornerstone of culinary traditions. Traditional production of animal foods such as fishmeal, soya, and cereals needs to be further intensified in terms of resource efficiency and extended using alternative sources [1,2]. By 2030, more than 9 billion people will need to be fed [3], along with the billions of animals raised annually for food or recreational purposes, such as pets. Furthermore, phenomena such as water and land pollution caused by intensive livestock production and deforestation caused by overgrazing will contribute to climate change and other destructive impacts on the environment. In recent years, the link between farming practices and environmental, animal, and human health has become increasingly evident, demonstrated, and defined in the One Health approach, which calls for solutions to be found to the challenges of our time [4]. One of the ways to address the problem of food and feed safety is through insect farming. Farmed insects are highly nutritious, rich in proteins, fats, and minerals, and can be raised using food waste as a feeding resource. Furthermore, they can be consumed whole or reduced to powders or pastes and incorporated into other types of food. Additionally, they can be processed into various forms for incorporation into animal feed [5].

Utilizing insects in nutrition and feed production offers numerous benefits, including high conversion efficiency and the ability to transform organic waste into valuable protein sources. This approach aligns with the principles of sustainability, circular economy, and resource optimization, offering a viable solution for poultry diets [6]. Insects live everywhere, reproduce quickly, have a high growth rate and food conversion rate, and have a low environmental impact throughout their life cycle.

The use of insects for nutrition and feed production presents various advantages for the environment, for human health, and for the improvement of the social conditions and livelihoods of various populations [7]. From a nutritional point of view, insect meals offer a superior amino acid profile compared to conventional feeds, reducing the need for synthetic additives and potentially lowering production costs. This can have not only an economic advantage but also a qualitative one, both directly on the welfare of the chickens (the feed must meet the amino acid and energy requirements of broilers for efficient growth and development) and indirectly on the quality of the meat obtained [8].

Environmentally, insects require fewer resources and emit fewer greenhouse gases compared to conventional livestock sources, contributing to sustainable agricultural practices [9]. Insects can feed on organic waste such as food remains and human products, compost, and animal sewage and can transform them into high-quality proteins that can in turn be used for animal feed.

Furthermore, addressing challenges related to food security and global protein demand is critical, and insect-derived meals provide a scalable, nutritionally dense solution that can supplement traditional feed sources. To this end, our research was carried out on samples of chicken fed with different types of feed (traditional, sustainable, live larvae, and dry larvae) to identify their volatile patterns and the characteristics of volatile organic compounds (VOCs) detected. In particular, traditional analytical methods such as solid-phase microextraction-gas chromatography-mass spectrometry (SPME-GC-MS) and innovative approaches such as electronic noses (E-noses) have been used. In many applications, the two technologies can be complementary. The SPME-GC-MS technique can be chosen for a detailed analysis of the VOCs, identifying and quantifying the chemical classes (such as amines, aldehydes, alcohols, esters, ketones, and others) and the specific compounds (such as hexanal, 1-octen-3ol, tridecane, and others) found in the samples of interest. However, the costs and the need for specialized knowledge make these traditional methods unsuitable for in-line or at-line use in meat processing facilities. Instead, electronic noses mimic the human olfactory system by detecting and analyzing the total pattern of volatile VOCs emitted by a biological sample or food product. The core of electronic nose (E-nose) systems is the gas sensors that cover many technical solutions, including metal oxide sensors (MOX). While these easy portable devices offer rapid, cost-effective, nondestructive analysis with minimal sample preparation and the ability to capture complex aroma profiles, on the other hand, their main disadvantage is that they are less precise. E-nose and, in particular, sensor arrays are not able to give the name of the molecules they are analyzing as output [10], but they can be employed for rapid screening, providing the total volatile fingerprint and allowing for a separation between the samples after multivariate statistical analysis (LDA or PCA). In addition, they can be trained to recognize target samples thanks to machine learning algorithms. This fact is very useful in the whole agri-food chain, from the field to the industry of food transformation and finally to the fork, since the volatile compounds influence consumer choices [11,12,13].

After nearly three decades of development, E-Nose technology has made great progress, and the efficacy of MOX for various applications in the complex food world has been investigated in numerous studies. For food quality control detection (e.g., freshness, adulteration, microbiological contamination, quality classification), data are reported among others in meat, fish, eggs, maize, potatoes, milk, and beer [14,15,16,17,18,19,20,21,22,23]. Other research is about monitoring the evolution of VOCs as a function of time (e.g., shelf life, aging, post-harvest storage, ripening, and fermentation) [24,25,26,27,28]; and about unmasking fraud on the origin of food and determining its real authenticity (e.g., geographical origin, plant cultivar, animal feed diet) [29,30,31].

In this study, the volatile components have been examined due to their susceptibility to various factors, notably the diet of animals engaged in food production. Differences in feed composition may lead to variances in the chemicals in meat, consequently affecting sensory characteristics [32] and creating unique volatile patterns.

In view of environmental sustainability, animal welfare and production, and human healthy alimentation, our research, using an integrated approach (an innovative S3+ device based on an array of six metal oxide semiconductor gas sensors and traditional analytical techniques—gas chromatography coupled with mass spectrometry (SPME-GC-MS)), not only highlights the nutritional potential of insect meals but also demonstrates the efficacy of sensor technology in monitoring food diversity and processing stages (raw and cooked), representing an advantageous decision-making aid to the business transformation process. Indeed, by optimizing feed composition and monitoring volatile compounds, poultry producers can enhance both the sustainability and safety of poultry production systems, contributing to a more efficient and environmentally friendly poultry industry.

## 2. Materials and Methods

### 2.1. Birds, Husbandry, and Diets

The in vivo trial took place at the poultry facility of the University of Turin in Italy, with the experimental protocol (No. 814715) receiving approval from the Bioethical Committee of the university. Adhering to European Union organic farming regulations (Regulation (CE) n. 834/2007), all birds experienced identical management and environmental conditions. At 39 days old, chicks were individually identified with wing marks and selected based on their average live weight (LW) of 316.8 ± 1.4 g. A total of 192 birds were then allocated to the experimental poultry facility, evenly distributed among 18 pens, each housing 8 birds and measuring 2.0 × 3.2 m, with rice hulls as litter. All birds had unrestricted access to an outdoor area of similar dimensions.

The birds were divided into three groups, with each group comprising 6 pens (considered replicates). The control group (CONTROL) received a basal diet containing conventional ingredients like soybean meal, while the ST group received an experimental basal diet where soybean meal was fully replaced by alternative ingredients. Two additional experimental groups were fed the same experimental basal diet as ST, with the inclusion of either dehydrated (DL) or live (LL) black soldier fly larvae (BSFL) at a level equal to 5% of the expected daily feed intake of dry matter (DM). Weekly, the feed consumption by the animals was calculated, and the amount of larvae provided was adjusted to supply 5% DM of the actual feed consumed. Diets were formulated to be isonitrogenous and isoenergetic, following INRA’s nutritional values for chickens (metabolizable energy, AME 11.8–11.9 MJ/kg; crude protein: 18.1%; ether extract: 3.59–3.63%; crude fiber: 3.28–4.80%). The feeding regimen details are outlined in Fiorilla et al. (2024) [33]. Dehydrated BSFL were supplied by “Entomo Agroindustrial” (Murcia, Spain), while live BSFL were sourced from “Inagro” (Rumbeke-Beitem, Belgium). Larvae were shipped weekly from Belgium and kept in a diapause state, reactivated before administration following the procedure outlined by Bellezza Oddon et al. (2021) [34].

The trial lasted for 135 days, starting from the chick age of 39 days until the final slaughter at 174 days. After a 12 h fasting period, chickens were electrically stunned following the standard regulations of the European Union (Council Regulation (EC) No 1099/2009 of 24 September 2009 [35]). The right cranial side breast filets were weighed, vacuum-packed, and frozen at −20 °C.

To investigate the volatile profiles of the poultry samples, two different techniques were performed simultaneously on the same sample: solid phase microextraction-gas chromatography-mass spectrometry (SPME-GC-MS) and electronic nose thanks to the recently described S3+ device.

### 2.2. Conditions for Stocking and Cooking Samples

All samples were stored at −20 °C before analysis to maintain their properties. Thawing methods varied depending on the intended use. Raw samples were thawed overnight at 4 °C in a refrigerator, while cooked samples were boiled directly from frozen. Boiling was conducted in a glass container filled with water on a hotplate equipped with a thermal probe, maintaining a temperature of 85 °C. Samples were cooked for 25 min while sealed in vacuum bags, then allowed to cool, sliced, and finally placed in the analysis box. Throughout each phase, sterility was ensured to prevent contamination and the detection of undesirable metabolites resulting from bacterial activity. Sterilized materials, Bunsen burners, or laminar flow cabinets were utilized, and work surfaces were sanitized to ensure optimal results.

In total, 48 samples from 2 different slaughters were analyzed. Each slaughter underwent 4 different diet treatments, with half of each treatment analyzed raw and the other half cooked. The diets that have been given to them, with the necessary codes entrusted, are listed below:

1. Traditional diet—code CONTROL;
2. Live larval integrated diet—code LL;
3. Dry larval integrated diet—code LD;
4. Sustainable diet—code ST.

Consequently, 3 samples from each treatment were analyzed raw, while 3 were cooked. For each sample, triplicate analyses were performed using the GC-MS instrument, along with 10 measurements on the S3+ device. In total, 624 analyses were conducted.

### 2.3. Preparation of GC-MS Samples

A quantity of 3 g of samples was extracted using a corer and transferred into 20 mL chromatographic vials equipped with aluminum caps and PTFE silicone septa. To ensure representative results, three different corers were employed for each vial, sampling from distinct points. Following closure, the samples were refrigerated at 4 °C until analysis to maintain the integrity of the volatilome profile and ensure consistent conditions for all analyses. Triplicate values were obtained for each sample using three separate vials to ensure statistically significant outcomes.

#### GC-MS Analysis Conditions

During analysis, a DVB/CAR/PDMS 50/30 μm solid-phase microextraction (SPME) fiber (Supelco Co., Bellefonte, PA, USA) was exposed to the vial headspace for 90 min at 60 °C to extract all volatile organic compounds (VOCs).

Chromatographic separation of analytes was performed using a Shimadzu GC 2020 (Kyoto, KYT, Japan) coupled with a Shimadzu MS-QP2020 mass spectrometer (Kyoto, KYT, Japan). The fiber was desorbed for 6 min into the GC injector port set in direct mode, where the entire sample was vaporized. The column temperature was maintained at 250 °C. The utilized column was a MEGA-5MS with dimensions of 25 m × 0.25 mm × 0.25 μm film thickness (Agilent Technologies, Santa Clara, CA, USA). The mass spectrometer operated in EI mode at 70 eV, with the ion source temperature set to 240 °C. Mass spectra were recorded in the range of 35 to 500 m/z in TIC mode with scanning intervals of 0.3 s. For all analyses, hydrogen (99.99%) generated by GENius PF500 (FullTech Instruments Srl, Rome, Italy) was used as the carrier gas. The selected pressure was 35.7 kPa, with a column flow of 2.2 mL/min, a linear velocity of 87.4 cm/s, and a purge flow of 4.0 mL/min. The detector temperature was set at 240 °C. The GC oven temperature program commenced at 40 °C, held for 1 min, followed by a temperature increase of 4.5 °C/min until reaching 50 °C. Subsequently, the temperature was increased at a rate of 6.5 °C/min to reach 80 °C. Finally, the temperature was ramped up to 15 °C/min to reach a final temperature of 180 °C. The total run time was 17 min, with a total analysis time, including exposure time, of 107 min. Peak identification was performed by comparison with three different mass spectra libraries: Nist11, Nist 11b, and FFNSC2. Chromatographic peaks were integrated into automatic mode using peak area as a parameter, considering at least 70 peaks with an area value not less than 500 AMU. Other parameters used in automatic peak integration included a slope of 100/min, a width of 2 s, a drift of 0/min, and a doubling time (T.DBL) of 1000 min. No smoothing method was applied. Finally, for post-run analysis, quantification of volatile compounds was expressed as relative abundance/time (% GC area) with mean ± standard deviation (st).

### 2.4. S3+ Samples Preparation

Sample preparation for S3+ analysis involved utilizing the remaining samples from GC-MS analysis, approximately 6 g in quantity. These samples were placed in a polypropylene (PP) box equipped with 2 holes on the cap to accommodate the positioning of aspiration tubes. After sealing the samples in the box, they were stored in a refrigerator to maintain standardized parameters and minimize product degradation.

#### 2.4.1. Calibration of MOX Sensor Arrays

The S3+ device and the sensor arrays used in this work were fully developed and optimized (details see Section 2.3) in collaboration with the NANO SENSOR SYSTEMS Srl spin-off of the University of Brescia, Italy. This device employs a gas array of metal-oxide sensors composed of various materials, specifically tailored for the target compounds [36]. The sensor calibration process adhered to rigorous protocols to ensure the reproducibility of the results. Initially, the sensor underwent annealing to facilitate stabilization of the sensing layer on the substrate. The annealing process was tailored according to the intended application of the sensor, with adjustable parameters including temperature (ranging from 500 °C to 800 °C) and duration (ranging from 1 h to 10 h). 

Subsequent to annealing, each sensor underwent an aging process in the air to standardize and reduce the electrical resistance (measured in ohms) of the sensing layers [37]. This aging period, adaptable to the sensitivity requirements of future applications, played a crucial role in optimizing sensor performance. The validation of the sensor was carried out within a well-established system comprising several components: a chamber with standardized dimensions to ensure uniform airflow, a mass flow program regulating the intake from air and ethanol pressure cylinders, and an electronic board overseeing sensor conditioning, monitoring, and data transmission to the cloud. The S3+ device consisted of a sensor chamber, a fluid dynamic circuit for the distribution of volatile compounds, and an electronic control system. Within the sensor steel chamber resided six in-house-developed Metal Oxide (MOX) sensors, each doped differently: two utilizing SnO_2_, two incorporating SnO_2_ with Pd, and two with SnO_2_ with Au. Operated at 500 °C (as outlined in Table 1), this temperature facilitated a clear delineation between sensors and their ambient environment.

The chamber dimensions were 11 × 6.5 × 1.3 cm, with sensor selection based on optimal performance during preliminary testing. Volatile compounds were constrained to pass only through inlet and outlet channels within the sensor chamber. The response of the MOX sensors relies on changes in electrical resistance caused by the interaction of volatile compounds with the sensor surface. This interaction leads to variations in charge carrier concentration, influencing the sensor’s conductance [38]. Environmental parameters such as temperature, humidity, and flow were continuously monitored within the chamber. The dynamic fluid circuit comprised a pump (Knf, model: NMP05B), polyurethane tubes, an electro valve (Camozzi Group s.p.a., model: K000-303-K11M), and a metal cylinder containing activated carbon. This circuit facilitated air filtration to prevent contamination by environmental odors, thereby ensuring the integrity of sensor responses. The solenoid valve positioned at the chamber inlet regulates pump flow with a maximum rate of 250 standard cubic centimeters per minute (sccm). The electronic board processed sensor responses, detecting changes in electrical resistance and controlling sensor temperature, a critical parameter for volatile compound detection. Ultimately, the system transmitted data to a dedicated web application for the S3 device via an internet connection, underscoring its status as an Internet of Things (IoT) device [39].

#### 2.4.2. S3+ Setup

**Figure 1:**
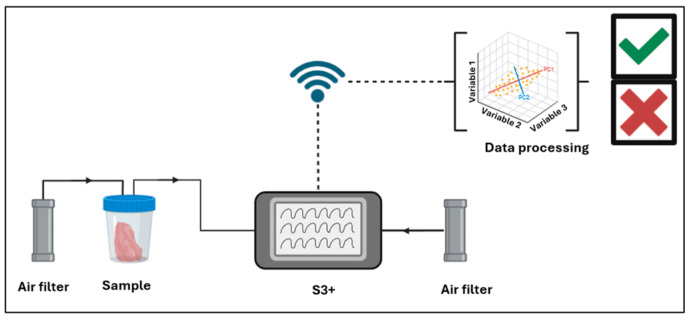
S3+ setup representation.

To conduct the analysis, the samples were placed in a 30 °C bath created using a hotplate equipped with a thermal probe, secured to prevent water infiltration, and connected to the instrument and carbon filters with PP tubes sealed by Parafilm^®^. Two carbon filters were utilized, one attached to the sample to filter air in the headspace and another attached to the S3+ device. Each analysis lasted 13 min, comprising 100 s for sensor stabilization, 200 s for sample analysis, and 500 s for sensor recovery. Ten replicates were conducted within 130 min. A schematic representation of the S3+ setup is provided in Figure 1.

**Figure 2:**
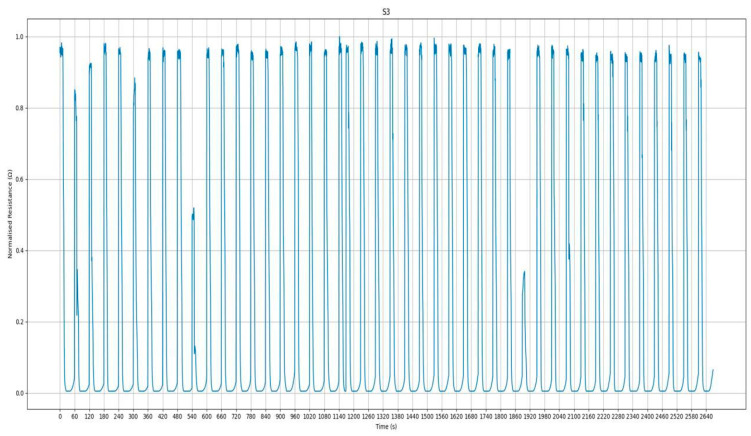
Graphical representation of the output of a single sensor. The *y*-axis shows the resistance value (Ω), while the *x*-axis shows time (s).

For each sample, 10 replicates were performed, analyzing sensor output (Figure 2) such as resistance, which was normalized to the initial acquisition value (R0). The difference between the initial value and the minimum value during analysis was calculated for each sensor.

Subsequently, the R/R0 parameter and standard deviation were determined for each sensor across all 10 measurements. The data from the various sensors were transmitted to the Microsoft Azure platform, where two web applications are available: a management portal and a mixture classification service. The output information from these sensing devices is interpreted using multivariate statistical analysis.

### 2.5. Post-Run Analysis

Linear Discriminant Analysis (LDA) is a technique used in statistics, pattern recognition, and machine learning to find a linear set of functions that distinguish between two or more classes of events [40]. The core concept of this method is to decrease the dimensionality of the data set while maintaining the significance of the T2 statistic, which assesses the hypothesis regarding the equality of means in a multidimensional context. Through LDA, a new space is generated where the dimensionality is reduced to, at most, k − 1 (where k represents the number of classes) (see Table 2) [41,42].

## 3. Results and Discussion

### 3.1. Poultry

In this study, both raw and cooked poultry samples, fed with different diets (CONTROL, LL, LD, and ST), were examined. Innovative techniques (electronic nose) and traditional techniques (GC-MS) have been used to conduct the research study. It is well known that raw meat has a bland flavor with little aroma. However, raw meat contains numerous precursors of meat flavor, which result in the formation of volatile odor compounds, especially during cooking [43].

### 3.2. GC-MS Detection Results

**Figure 3:**
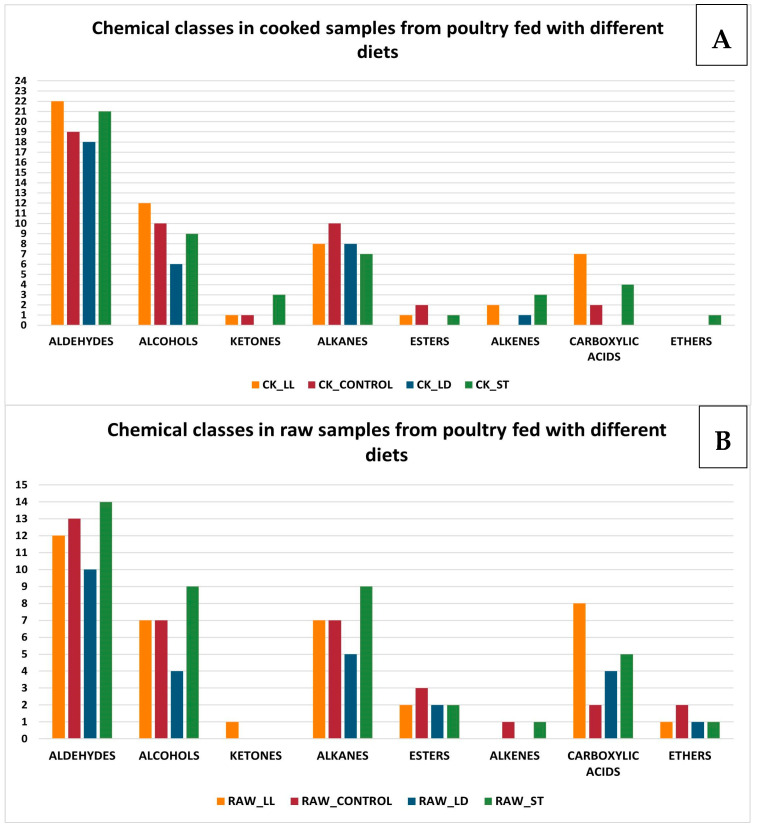
(**A,B**) Chemical classes in cooked and raw samples from poultry fed with different diets.

The results obtained from the SPME-GC-MS analysis allowed for the identification of the volatile profile of each sample. A meticulous examination of these results revealed a variety of compounds, some of which are extremely important for the aromatic impact of the product. The chemical classes identified in the analyzed poultry samples include aldehydes, alcohols, ketones, alkanes, esters, alkenes, carboxylic acids, and ethers. Among these, aldehydes, alcohols, alkenes, and carboxylic acids were the most prevalent compounds found in both raw and cooked poultry samples, irrespective of the diet provided (Figure 3A,B).

From the above images, it is evident that there is a variation in chemical compounds between raw and cooked poultry products. Specifically, there is a quantitative reduction in aldehydes, alcohols, and ketones in raw samples in comparison to cooked samples (except alcohols in sample CK_ST and RAW_ST, which are 9 in both, and ketones in sample CK_LL and RAW_LL, which remain 1), an increase in esters, carboxylic acids (except carboxylic acids in sample CK_CONTROL and RAW_CONTROL, which are 2 in both), and ethers, while alkanes and alkenes remain nearly unchanged. This behavior is primarily due to two factors: cooking processes and variations in specific compounds [44].

Regarding cooking processes, it is noted that high temperatures lead to protein denaturation, lipid oxidation, the Maillard reaction, and Strecker degradation. The detailed explanations are as follows:

1. Protein Denaturation: cooking causes the denaturation of proteins, which alters the structure of protein molecules, leading to the formation of new volatile compounds.
2. Maillard Reactions: this set of chemical reactions occurs between amino acids and reducing sugars at elevated temperatures, producing a wide range of volatile compounds responsible for the flavor and aroma of cooked food.
3. Related to the Maillard reaction is a subset of chemical processes called Strecker degradation, which plays a critical role in meat flavor generation and, in general, in producing aroma-active volatiles in processed foods. The Strecker degradation converts an α-amino acid into an aldehyde containing the side chain by way of an imine intermediate, directing the Maillard reaction from chromogenic pathways toward more aromagenic pathways [45]. Depending on the parent amino acid, the Strecker aldehydes normally have low-odor threshold values with characteristic aroma properties.
4. Lipid Oxidation: lipids present in the meat oxidize during cooking, producing aldehydes, alcohols, and ketones in the initial stages, which subsequently transform into acids and other compounds [46].

The variation in specific compounds between raw and cooked poultry samples [47] is explained as follows:

- Reduction in aldehydes, alcohols, and ketones: aldehydes can further oxidize during cooking, transforming into carboxylic acids. Alcohols, in turn, can oxidize into aldehydes and ketones, which can subsequently transform into carboxylic acids. Ketones can undergo further oxidation reactions, becoming carboxylic acids.
- Increase in esters, carboxylic acids, and ethers: esters can form through reactions between alcohols and acids, facilitated by the presence of heat. Carboxylic acids increase due to the oxidation of aldehydes, alcohols, and ketones. Ethers can form through condensation reactions between alcohols, especially under high heat conditions.
- Unchanged alkanes and alkenes: these hydrocarbon compounds are relatively stable and do not readily participate in the chemical reactions that occur during cooking. Therefore, their concentrations tend to remain unchanged.

**Figure 4:**
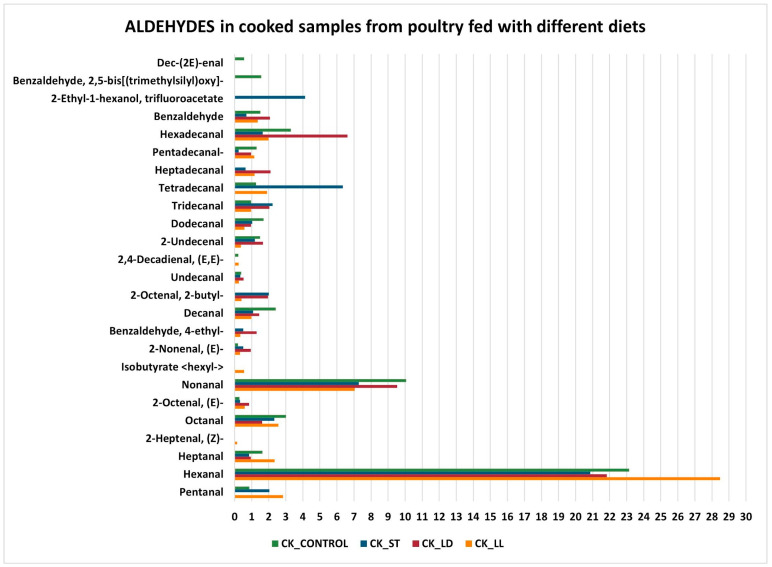
Aldehydes in cooked samples.

**Figure 5:**
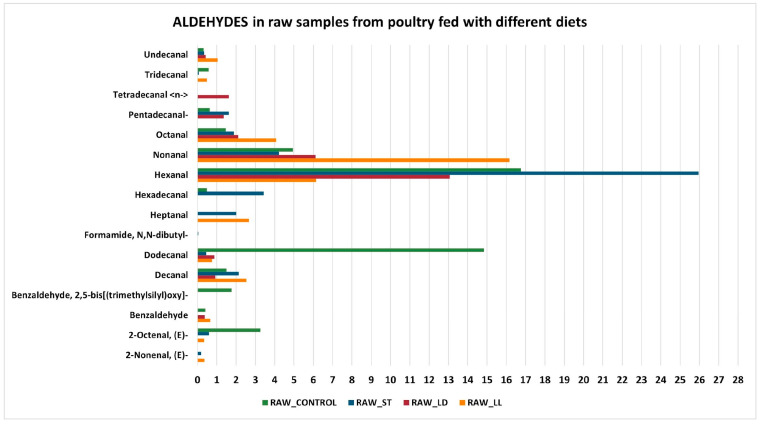
Aldehydes in raw samples.

In summary, cooking induces a series of chemical reactions that transform the compounds present in raw poultry products. Oxidation reactions and chemical transformations lead to a reduction in aldehydes, alcohols, and ketones, while increasing carboxylic acids, esters, and ethers. Alkanes and alkenes remain unchanged due to their chemical stability. A variation in chemical compounds is also evident when comparing the different diets (LL, CONTROL, LD, and ST). The following graphs examine specific chemical compounds (Figure 4 and Figure 5): the *y*-axis represents the volatile molecules found in the examined samples, while the *x*-axis shows the mean percentage abundance, indicating the significance of each compound for the specific sample. Regarding aldehydes, hexanal (C_6_H_12_O) is the most prominent molecule in all samples. It is an aliphatic aldehyde naturally found in many foods and plant products. Hexanal can be produced through both the lipoxygenase pathway, which involves the enzymatic oxidation of polyunsaturated fatty acids, and the non-enzymatic chemical oxidation of lipids. Both pathways are crucial for the formation of volatile compounds that contribute to the aroma and flavor of many foods, as well as serving as indicators of freshness and quality [48]. Moreover, aldehydes dominate in the headspace of cooked meat, while hexanal is a powerful contributor to the odor of chicken meat due to its low odor threshold [49]. In particular, hexanal provides a green and fresh aroma.

**Figure 6:**
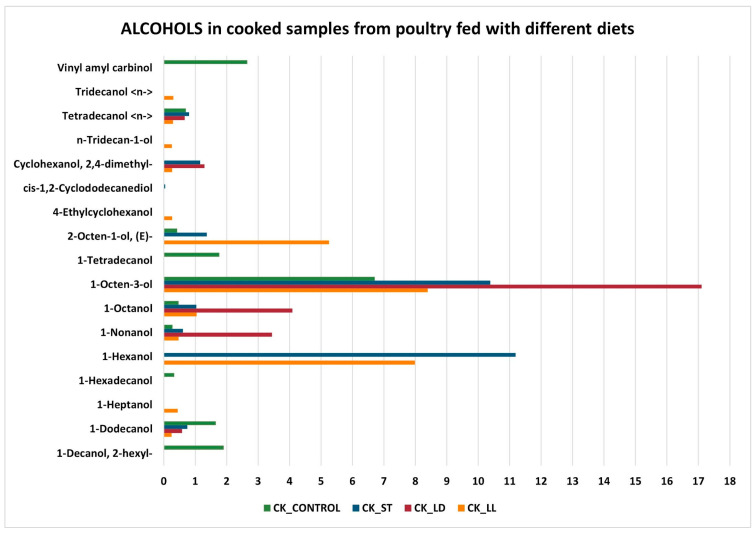
Alcohols in cooked samples.

**Figure 7:**
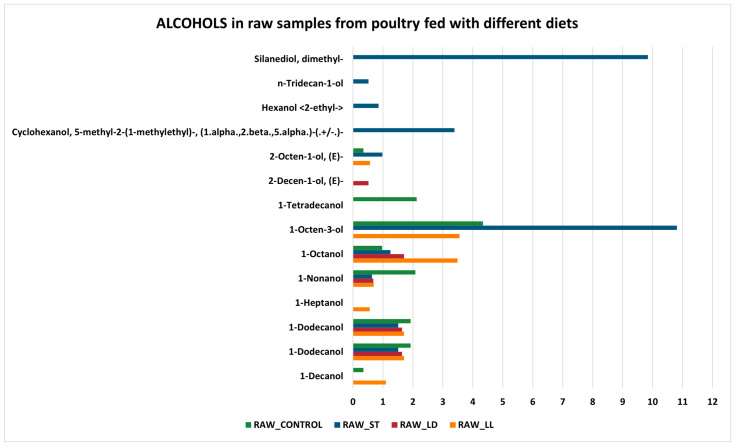
Alcohols in raw samples.

Regarding alcohols (Figure 6 and Figure 7), 1-Octen-3-ol is the compound with the highest average abundance in all cooked and raw samples (except for RAW_LD, which does not have one). It is an organic compound with a characteristic fungal odor, naturally present in various food products, and used in multiple applications. It is generally produced by various organisms through the enzymatic decomposition of unsaturated fatty acids. In addition, 1-octen-3-ol concentration is found to be differentiated mainly at high temperatures and could be a marker of lipid oxidation but not microbial spoilage [50]. However, alcohols, as a whole, could have an insignificant contribution to odor due to their relatively high odor threshold values [51]. It is well known that volatiles originating from lipid oxidation, such as alcohols and ketones, have high odor thresholds (mg/L range), aldehydes (µg/L to mg/L range), and N- and S-heterocyclic compounds originating from the Maillard reaction and Strecker degradation have odor thresholds in the µg/L range.

Both in cooked and raw samples, there are some compounds that are present only in one type of diet. In particular, in cooked samples, there are nine alcohols on a total of seventeen compounds revealed (Vinyl amyl carbinol in CK_CONTROL; Tridecanol <n-> in CK_LL; n-Tridecan-1-ol in CK_LL; cis-1,2-Cyclododecanediol in CK_ST; 4-Ethylcyclohexanol in CK_LL; 1-Tetradecanol in CK_CONROL; 1-Hexadecanol in CK_CONTROL; 1-Heptanol in CK_LL; 1-Decanol, 2-hexyl- in CK_CONTROL), while in raw ones the half of samples (seven on fourteen measured) are resulted present only in a type of diet [Cyclohexanol,5-methyl-2-(1-methylethyl)-(1.alpha.,2.beta.,5.alpha.)-(.+/−.)-; Hexanol <2-ethyl->; n-Tridecan-1-ol, Silanediol, dimethyl- all three present only in RAW_ST.

**Figure 8:**
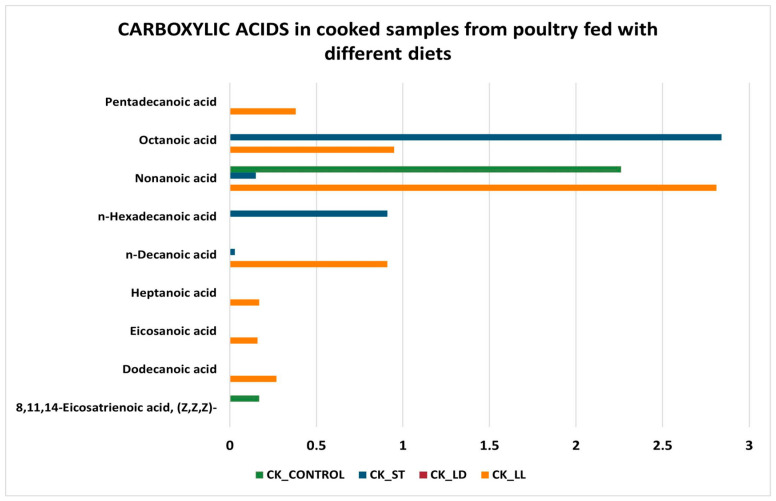
Carboxylic acids in cooked samples.

**Figure 9:**
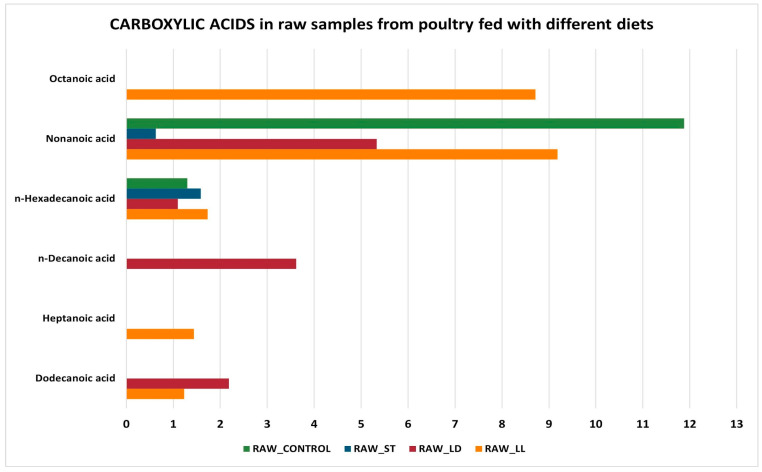
Carboxylic acids in raw samples.

Regarding carboxylic acids (Figure 8 and Figure 9), the samples cooked with the LS diet do not have any compounds. Conversely, CK_LL and CK_ST samples are those with more carboxylic acids, especially nonanoic acid and octanoic acid (medium-chain fatty acids that contribute to the flavor and aroma of chicken meat). In raw samples, carboxylic acids tend to increase significantly, especially for raw samples fed on the LD diet (RAW_LD). The increase in carboxylic acids in raw poultry meat compared to cooked meat can be explained by several reasons related to chemical processes that occur during cooking [52]:

- Thermal decomposition: during cooking, fatty acids can undergo thermal decomposition, leading to the formation of volatile compounds or the degradation of shorter-chain fatty acids. This can reduce the concentration of nonanoic acid and octanoic acid in cooked meat.
- Oxidation: cooking, especially at high temperatures, can accelerate the oxidation of fatty acids. Nonanoic and octanoic acids can oxidize and transform into other compounds, such as aldehydes, ketones, and shorter acids, thereby reducing their concentration in cooked meat.
- Evaporation: some short- and medium-chain fatty acids can evaporate during cooking. Octanoic acid and nonanoic acid have relatively low boiling points compared to longer-chain fatty acids, which can lead to their partial loss as volatile compounds during cooking.
- Maillard reactions: during cooking, especially at high temperatures, Maillard reactions occur, which are complex chemical reactions between amino acids and reducing sugars. These reactions can influence the lipid profile of the meat, leading to the formation of new compounds and the reduction of the originally present fatty acids.
- Lipolysis: in the case of raw meat, enzymatic processes such as lipolysis can be active, leading to the release of free fatty acids like nonanoic acid and octanoic acid. Cooking inactivates these enzymes, interrupting the lipolysis process and thereby reducing the formation of free fatty acids.

These combined factors explain why levels of acids can be higher in raw chicken meat and decrease after cooking. Several acids were detected in other studies on different meat species, such as nonanoic acid in goat and fried chicken meat [53], pentanoic acid in roasted chicken [54], and octanoic acid in roasted chicken [55].

**Figure 10:**
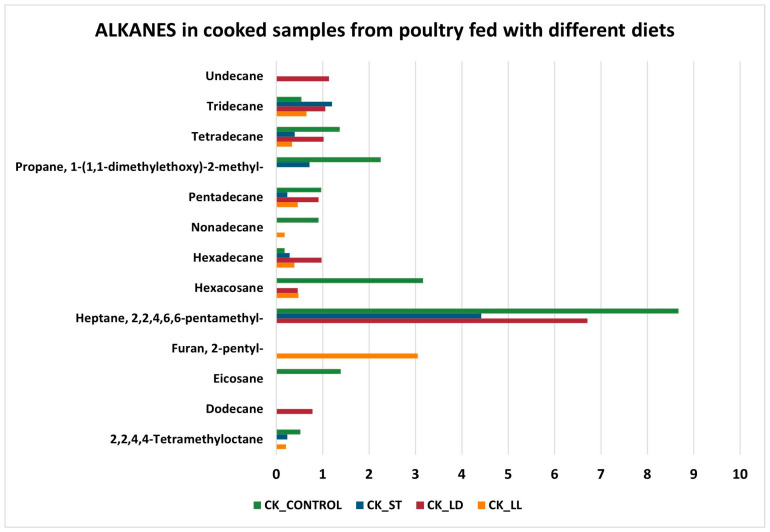
Alkanes in cooked samples.

**Figure 11:**
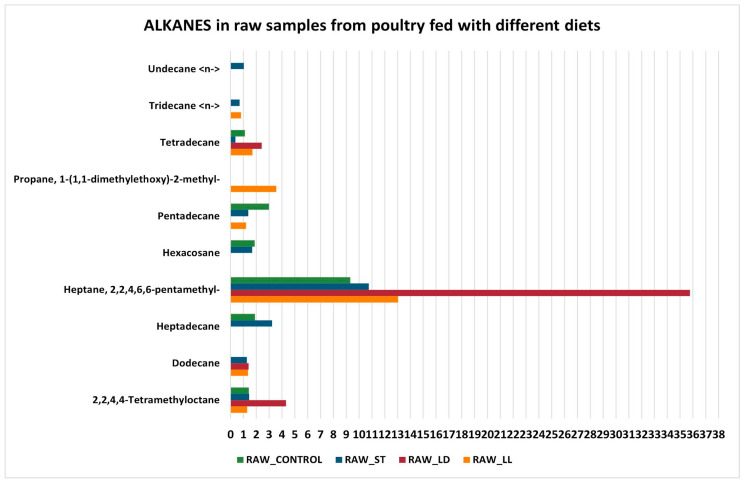
Alkanes in raw samples.

Another relevant chemical class is that of alkanes (Figure 10 and Figure 11). Heptane, 2,2,4,6,6-pentamethyl, is the compound with a very high impact in all cooked and raw samples. Although hydrocarbons are not primarily responsible for the aroma, they can still affect the organoleptic characteristics of the meat, contributing to a complex and unique aromatic profile [56,57].

Esters, ethers, and ketones were found in small quantities in all the samples analyzed, and their average abundance is below 1%. This means that their contribution to the aromatic profile is irrelevant. An exception is the ketone compounds 2-heptanone, 3-octanone, and 2-nonanone, which have an average abundance of 1.5%, 4.5%, and 2.3%, respectively, in samples cooked with the ST diet. These compounds are correlated with spoilage since they are metabolic products of several microorganisms found in meat, such as *Pseudomonas* spp., *Carnobacterium* spp., and Enterobacteriaceae.

### 3.3. S3+ Detection Results

Having performed the GC-MS analysis to highlight the differences in the volatile fingerprints of poultry samples, we decided to investigate the same with the Small Sensor System (S3+). The device successfully analyzed the volatile fingerprints, allowing us to observe a separation between the samples, as shown in the LDA plot. The primary objective of LDA is to maximize the separation between classes while simultaneously minimizing the variance within each class [53]. In our case, it was used to reduce the dimensionality of the data collected by the S3+ device and to visualize the separation between samples fed with different diets.

**Figure 12:**
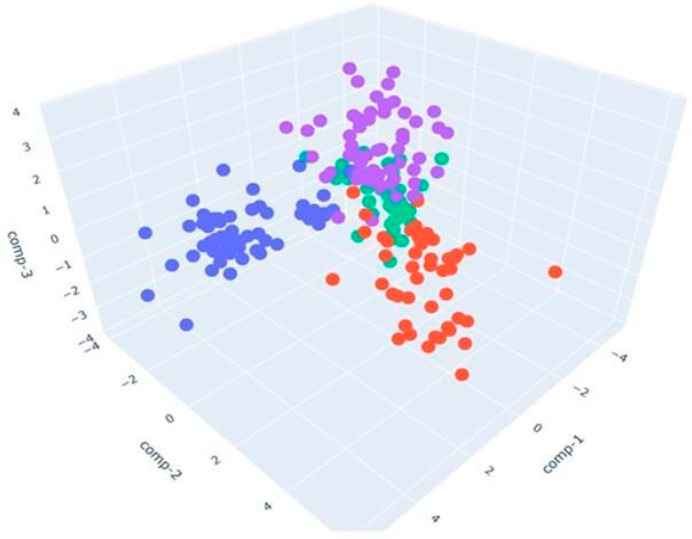
LDA in 3D, representing cooked samples with CONTROL diet (blue points), ST (red points), LD diet (green points), and LL diet (purple points).

Figure 12 presents a three-dimensional representation of the LDA applied to cooked poultry samples fed with different diets:

- The blue points represent the control samples (CK_CONTROL). The points appear clustered in a specific area, indicating that the control samples tend to have similar olfactory profiles.
- The red points indicate the samples with a sustainable diet (CK_ST). These points are located in an area distinct from the area of the blue dots, indicating significant differences in olfactory profiles compared to the control samples. The separate position suggests that a sustainable diet has a measurable and distinct impact on the olfactory profile of chicken meat.
- The green points represent the samples fed with a dry larval diet (CK_LD), and they are mainly concentrated in the center of the chart.
- The purple points correspond to the samples fed with a live larval diet (CK_LL). Purple dots are placed in their region of the LDA analysis space. Their separation from other groups implies that the diet based on live larvae has a complex and characteristic volatilome.

The calculation of LDA performances led to an accuracy of 86.53%.

**Figure 13:**
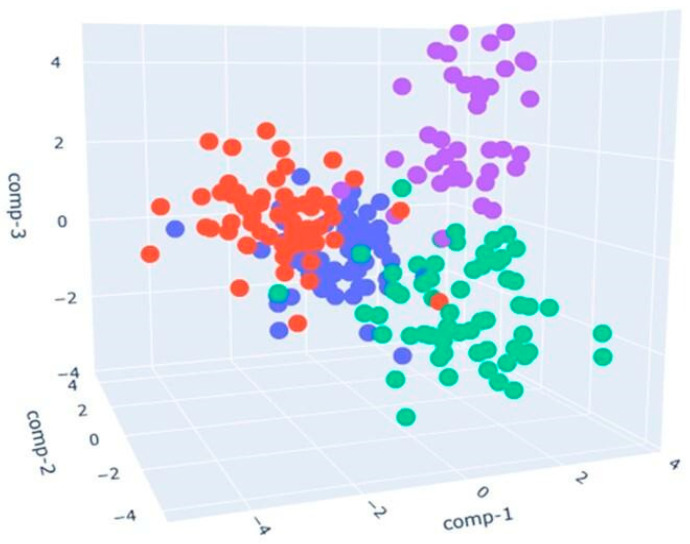
LDA in 3D, representing raw samples with the CONTROL diet (blue points), ST diet (red points), LD diet (green points), and LL diet (purple points).

Figure 13 presents a three-dimensional representation of the LDA applied to raw poultry samples fed with different diets:

- The blue points represent the control samples (RAW_CONTROL) and are mainly concentrated in the center of the chart.
- The red points indicate the samples with a sustainable diet (RAW_ST). These points are also concentrated in the center, partially overlapping with the blue control samples.
- The green points represent the samples fed with a dry larval diet (RAW_LD), primarily distributed on the lower right side of the chart.
- The purple points correspond to the samples fed with a live larval diet (RAW_LL), mainly distributed on the upper right side of the chart.

The RAW_LD and RAW_LL samples (green and purple) show good spatial separation from the other two groups (blue and red), indicating that the LDA effectively distinguished these samples based on their characteristics detected by the S3+. The control samples and those with a sustainable diet show significant overlap, suggesting they may have very similar characteristics as detected by the device.

In this case, LDA achieves a classification rate equal to 81.78%.

### 3.4. Confusion Matrix and ROC Curve Images

The results obtained with the S3+ device for generating the confusion matrix and ROC curve were divided using 80% of the total data for the training set and 20% for the test set. Regarding the raw samples, the value used for calculating the test set corresponds to 46, which is 20% of the total 228 samples. Similarly, for the cooked samples, the corresponding value is 42, which is 20% of the total of 209 samples.

#### 3.4.1. Confusion Matrix

**Figure 14:**
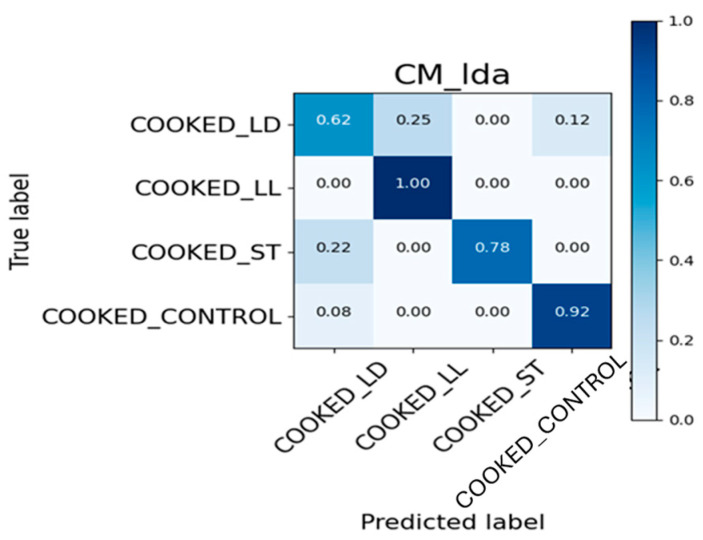
Confusion Matrix on cooked samples.

The confusion matrix, presented in Figure 14, is an effective method for evaluating the performance of a classifier on a test dataset with known true labels. In this case, the classifier was used to distinguish between four classes: COOKED_LD, COOKED_LL, COOKED_ST, and COOKED_CONTROL.

**Figure 15:**
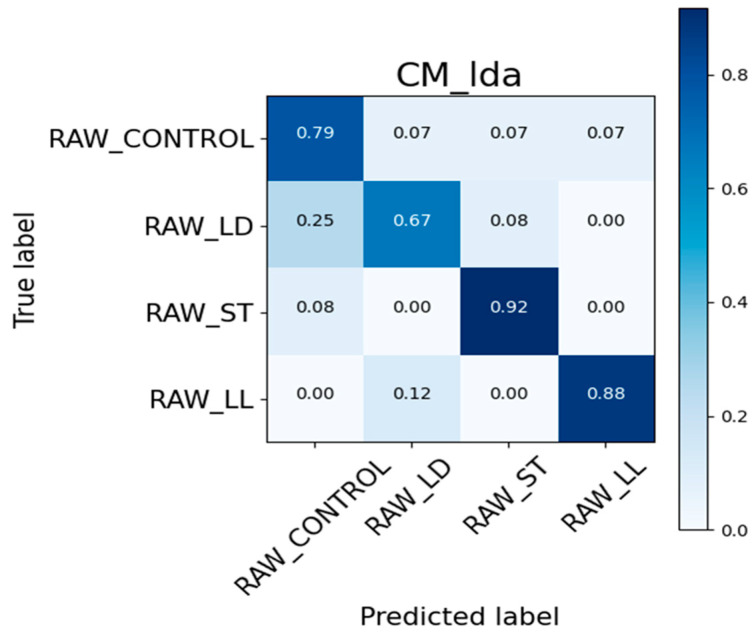
Confusion Matrix on RAW samples.

For the COOKED_LD class, the classifier correctly identifies 62% of the samples, but 25% are misclassified as COOKED_LL and 12% as COOKED_CONTROL. This indicates some degree of confusion between these categories. In contrast, the COOKED_LL class shows perfect accuracy, with 100% of its samples being correctly identified, highlighting high precision for this class. Considering the COOKED_ST class, the classifier achieves 78% accuracy. However, there is a notable error rate, with 22% of the COOKED_ST samples being incorrectly classified as COOKED_LD. For the COOKED_CONTROL class, the classifier performs well, with 92% of the samples correctly classified. There is a small error rate, with 8% of the COOKED_CONTROL samples being misclassified as COOKED_LD. Figure 15 provides a similar analysis for a different set of data: RAW_CONTROL, RAW_LD, RAW_ST, and RAW_LL.

The confusion matrix for the RAW data reveals several key observations about the classifier’s performance. For the RAW_CONTROL class, the classifier correctly identifies 79% of the samples. However, there is a 7% error rate, with these samples being misclassified as RAW_LD, RAW_ST, and RAW_LL. Examining the RAW_LD class, 67% of the samples are accurately classified, but there is a significant error rate: 25% of RAW_LD samples are misclassified as RAW_CONTROL and 8% as RAW_ST. The RAW_ST class demonstrates a high accuracy rate, with 92% of samples correctly classified. However, there is still an 8% error rate, with these samples being incorrectly identified as RAW_CONTROL. Similarly, the RAW_LL class shows a high accuracy rate of 88%, though 12% of these samples are misclassified as RAW_LD. The confusion matrix for the RAW data shows high accuracy for the RAW_ST and RAW_LL classes, while the RAW_LD class shows more confusion, particularly with RAW_CONTROL.

#### 3.4.2. ROC Curve

**Figure 16:**
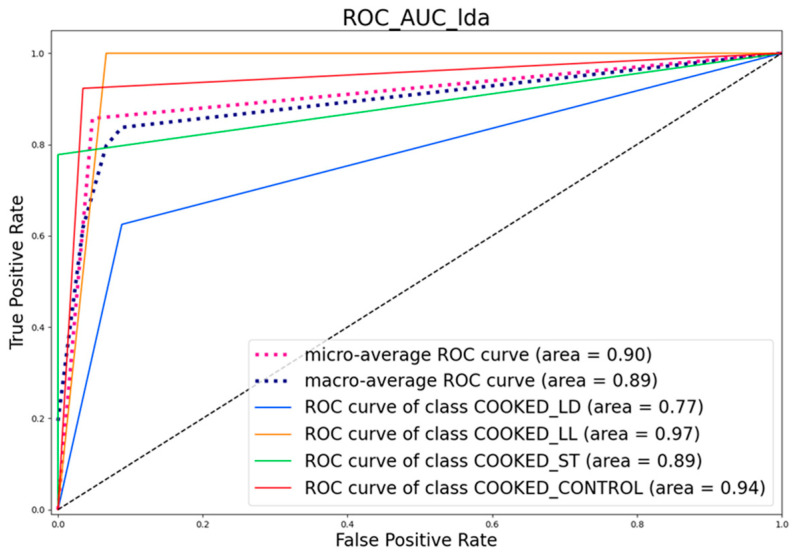
ROC Curve of cooked samples.

**Figure 17:**
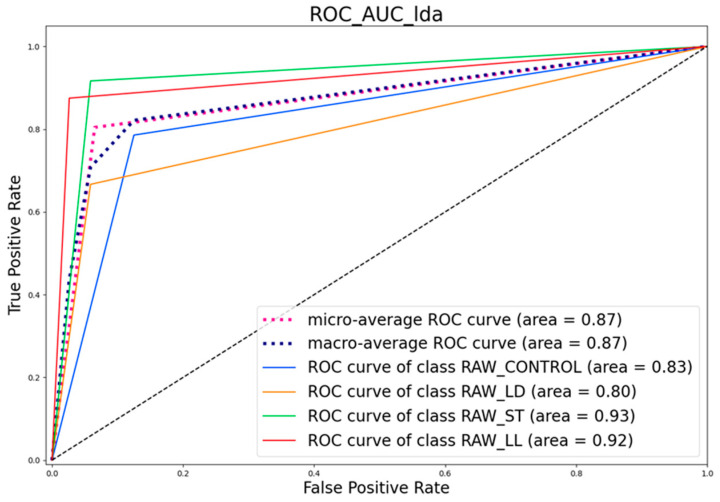
ROC Curve of raw samples.

Figure 16 and Figure 17 present the Receiver Operating Characteristic (ROC) curves for the different classes, along with the Area Under the Curve (AUC) values.

The black dashed line is the Random Classifier which is a versatile classification tool that makes an aggregated prediction using a group of decision trees trained using the bootstrap method with extra randomness while growing trees by searching for the best features among a randomly selected feature subset.

The ROC curve analysis provides several important insights into the classifier’s performance. The micro-average ROC curve has an AUC of 0.90, representing the average performance of the classifier across all classes, indicating good discriminative ability. Similarly, the macro-average ROC curve, with an AUC of 0.89, reflects the average performance considered independently for each class, also suggesting good overall performance. The COOKED_LD class has an AUC of 0.77, which indicates moderate performance but is lower compared to the other classes. The COOKED_LL class shows excellent discriminative ability with an AUC of 0.97. The COOKED_ST class has an AUC of 0.89, indicating good discriminative ability, while the COOKED_CONTROL class has an AUC of 0.94, reflecting very good discriminative ability. The ROC curves highlight that the classifier performs best for the COOKED_LL and COOKED_CONTROL classes, while the COOKED_LD class shows relatively lower performance compared to the others.

As for the raw samples, the micro-average ROC curve has an AUC of 0.87, representing the average performance of the classifier across all classes, which indicates good discriminative ability. Similarly, the macro-average ROC curve, with an AUC of 0.87, reflects the average performance when considered independently for each class, also suggesting good overall performance.

Looking at specific classes, the RAW_CONTROL class has an AUC of 0.83, indicating moderate performance. The RAW_LD class shows slightly lower performance with an AUC of 0.80, still within the moderate range. The RAW_ST class stands out with excellent discriminative ability, having an AUC of 0.93. The RAW_LL class also performs very well, with an AUC of 0.92, indicating very good discriminative ability.

The ROC curves for the RAW data highlight that the classifier performs best for the RAW_ST and RAW_LL classes, while the RAW_LD and RAW_CONTROL classes show relatively lower performance.

The images provide a detailed analysis of the model’s classification performance on both COOKED and RAW datasets. The confusion matrices indicate that the model has good precision for the COOKED_LL, COOKED_CONTROL, RAW_ST, and RAW_LL classes. The ROC curves confirm these observations, showing high discriminative ability for COOKED_LL, COOKED_CONTROL, RAW_ST, and RAW_LL, and relatively lower performance for COOKED_LD and RAW_LD. This information is crucial for understanding the strengths and areas for improvement of the model.

## 4. Conclusions

In conclusion, our study has demonstrated the importance of exploring alternative feeds for livestock intended for human consumption. The research focused on the analysis of volatile components in cooked and raw chicken samples fed with different types of feed: traditional, alternative diet, a diet with the addition of live larvae, and a diet with the addition of dried larvae. The study was conducted using innovative techniques (S3+) and traditional methods (GC-MS). This combined approach facilitated the classification and quantification of various gas samples, while the sole use of the S3+ device has proven to be a useful and effective tool for ensuring discrimination not only between cooked and raw samples but also among samples subjected to different diets. The integration of innovative feeds, such as those based on larvae, could represent a viable strategy to enhance the quality and sustainability of poultry production systems, contributing to a more efficient and environmentally friendly poultry industry. The effectiveness of LDA in this context underscores its potential as a powerful analytical tool for distinguishing complex gas mixtures, thereby highlighting its applicability in advanced volatile analysis and sensor technology development. By implementing continuous, non-destructive monitoring of the process, data can be consistently collected and utilized to develop an IoT-integrated system capable of managing the entire production process. This study is important for paving the way towards novel future applications in this field.

## Figures and Tables

**Table 1:** Schematic description of the setup for different sensing elements.

Type of Sensor	Doping	Working Temperature (°C)
MOX sensor	SnO_2_	500 °C 500°C
MOX sensor MOX sensor	SnO_2_ + Pd SnO_2_ + Au	500°C

**Table 2:** Features extracted from the recorder tracks of each sensor.

Features	Description
Sharpe Forward 25%	Variability index equivalent to the ratio between the mean and the standard deviation calculated from the beginning of the signal to 25% of it.
Sharpe Back 25%	Variability index equivalent to the ratio between the mean and the standard deviation calculated from the end of the signal to 25% of it.
Sharpe Forward 50%	Variability index equivalent to the ratio between the mean and the standard deviation calculated from the beginning of the signal to 50% of it.
Sharpe back 50%	Variability index equivalent to the ratio between the mean and the standard deviation calculated from the end of the signal to 50% of it.
Minimum derivative	Calculation of the minimum derivative of the function in the selected interval.
Maximum derivative	Calculation of the maximum derivative of the function in the selected interval.
Integral	Calculation of the integral of the function in the selected interval.
∆R	Often called excursion range, this feature represents the difference between the maximum and minimum values observed in the time series.
Logarithm of sum	The sum of the natural of the signal.
Minimum	The minimum value observed in the time series.
Maximum	The maximum value observed in the time series.

## Data Availability

Data are contained within the article.
